# Barriers and facilitators to antiretroviral therapy adherence among Peruvian adolescents living with HIV: A qualitative study

**DOI:** 10.1371/journal.pone.0192791

**Published:** 2018-02-15

**Authors:** Jerome T. Galea, Milagros Wong, Maribel Muñoz, Emiliano Valle, Segundo R. Leon, Dayana Díaz Perez, Lenka Kolevic, Molly Franke

**Affiliations:** 1 Department of Global Health and Social Medicine, Harvard Medical School, Boston, Massachusetts, United States of America; 2 Socios En Salud, Lima, Peru; 3 Instituto Nacional de Salud del Niño, Lima, Peru; Boston University, UNITED STATES

## Abstract

AIDS deaths among adolescents are increasing globally. This qualitative study investigated the barriers and facilitators to cART adherence among Peruvian adolescents living with HIV. Guided by a social ecological model, we analyzed transcripts from 24 psychosocial support groups for HIV-positive adolescents aged 13–17 years and 15 individual, in-depth interviews with cART providers and caregivers to identify the barriers and facilitators to cART adherence at the individual, family/caregiver and hospital levels. Most barriers and facilitators to cART adherence clustered at the individual and family/caregiver levels, centering on support provided to adolescents; history of declining health due to suboptimal cART adherence; side effects from antiretroviral drugs; and cART misinformation. Interventions to support adolescent HIV cART adherence should begin at the individual and family/caregiver levels and include an educational component. No adolescent living with HIV should die from AIDS in an era of accessible cART.

## Introduction

Despite a continuing global trend of fewer AIDS-related deaths in children and adults, the opposite has occurred among adolescents aged 10–19 years, with mortality rising from 18,000 deaths per annum in 2010 to 41,000 deaths in 2015 [1]. AIDS is now the leading cause of death among teenagers in sub-Saharan Africa [2] and UNAIDS estimates that 1.8 million adolescents 10–19 years of age are living with HIV worldwide [1]. But the success of modern HIV antiretroviral therapy—now widely available and able to transform HIV disease into a chronic and manageable illness—also presents new challenges as perinatally infected children enter adolescence [3].

Mortality from AIDS among persons with HIV disease who have access to combination antiretroviral regimens (cART) should be a rare event among all age groups, but compared to adults and children alike, adolescents are less likely to achieve viral suppression, which can eventually lead to treatment failure [4–6]. A recent meta-analysis of 50 publications with data from 53 countries and 10,725 adolescents and young adults found that 62% of adolescents taking HIV cART reached “adequate” adherence (defined by more than 85% of the medication taken correctly or viral suppression [7]). Notably, the lowest average adolescent ART adherences observed in that study were in North America [53% (95% CI 46–59; I^2^: 91%)], Europe [62% (95% CI 51–73; I^2^: 97%)] and South America [63% (95% CI 47–77; I^2^: 85%)] [7].

These findings raise important questions regarding factors impeding cART adherence, and interventions to address them. Especially for adolescents infected at birth, whom UNICEF notes as particularly vulnerable to AIDS mortality despite having spent a decade or more with successful HIV cART [6], the loss of health and potentially life signals a major gap in the HIV care continuum for this population. Complicating matters, many perinatally-infected adolescents will have initiated cART well after birth (and with sub-optimal regimens), presenting for care with severe HIV disease. These adolescents may face a number of chronic clinical complications causing severe morbidity due to the effect of HIV on an underdeveloped immune system [8].

A growing body of research over the past decade has begun to characterize the barriers and facilitators to cART adherence among adolescents, albeit primarily in either high-resource settings such as Europe [9], the United States [10] or in Sub-Saharan Africa [11–14]. In a multinational review of cART in perinatally HIV infected adolescents, Agwu and Fairlie consolidate adherence barriers to six primary domains: structural (e.g., access to healthcare; problems with school, housing, or work; and lack of transportation to clinic visits); patient-related (e.g., cognitive, emotional, behavioral, and life stage issues); provider-related (e.g., lack of multidisciplinary teams experienced in adolescent health); medication-related (e.g., treatment fatigue, complex regimens involving high pill burden and dosing frequency; and unpalatability of cART); disease-related (e.g. comorbid diseases like tuberculosis and malaria, especially in resource limited settings); and psychological factors (depression and psychological distress) [15]. Despite the wide applicability of these findings across settings, interventions to address them have been elusive and no gold standard exists. Reisner and colleagues point out in their review of cART adherence interventions for youth that a contextualized understanding of the factors associated with non-adherence, including multi-level social analysis, is an essential step in designing suitable interventions [16].

Social ecological systems theory [17] facilitates multi-level social analysis and could expand an understanding of the factors related to adolescent cART adherence and help identify areas most amenable to intervention. The theory as it relates to health posits that disease cannot be viewed exclusively at the individual level but as a reciprocal interplay between an individual and her/his environment at multiple levels or systems, such as family, friends, social networks, communities and culture [18]. Importantly, social ecological systems theory and the resultant social ecological models (SEM) do not exempt individual agency but rather view it as affecting and being affected by other factors in one’s system or ecosystem. SEM have been used widely to understand a diversity of health issues including adolescent HIV cART adherence [19].

In Latin America and the Caribbean, an estimated 74,000 10–19 year-olds are living with HIV [1]. Unfortunately, limited data on cART adherence and viral suppression rates hamper the ability to fully characterize the success of HIV care programs. But a 2015 review of the HIV continuum of care in Latin America reported the proportion of people living with HIV/AIDS with an undetectable viral load ranging from 12% in Venezuela to 25% in Mexico [20]; data specific to adolescent populations are unavailable. In response to the paucity of cART adherence information and interventions tailored to adolescents in Latin America, we used qualitative methods guided by social ecological systems theory to understand the factors related to cART adherence among HIV-positive Peruvian adolescents.

## Methods

### Participants and procedures

Data were collected from three different sources in Lima, Peru: psychosocial support groups for adolescents receiving HIV-cART; professional staff delivering care to adolescents taking HIV cART; and parents/caregivers of adolescents taking HIV cART.

The adolescents group was comprised of boys and girls aged 13 to 17 years old, all of whom were receiving their HIV care at a large urban hospital in Lima Peru. Adolescents at high risk of or with a history of suboptimal cART adherence (as identified by their treating physician) were invited to participate in one of two multi-session psychosocial support groups, according to their age: one for 13–15 year-olds and the other for 16–17 year-olds. The groups, which were conducted in a private space located at a hospital in Lima, were facilitated by women living with HIV (henceforth “peer facilitators”) who were experienced in conducting psychosocial support groups for HIV-positive persons. The groups met twice every month for 90 minutes on average over six months (12 sessions in total) and were audio recorded. Rather than follow a structured script or traditional focus group format where participants are typically asked to respond to specific, pre-determined questions, group content was decided spontaneously by the adolescents during the sessions. The peer facilitator’s role was to assist in the exploration of emergent group themes, encourage group participation, and assure mutual respect and other group norms (e.g. confidentiality, punctuality, etc.). Emphasis was placed on developing and nurturing mutual support among persons confronting similar or shared experiences.

In addition to the groups, individual, in-depth interviews were conducted with a physician, nurse, psychologist, administrative staff person and a peer counselor (a personal openly living with HIV), all employed by the Peruvian Ministry of Health’s HIV prevention and treatment program, as well as 10 parents/caregivers of adolescents receiving cART. The professionals were selected because of their experience in working with adolescents receiving cART for HIV and the perspectives they could have regarding cART adherence among this population while the parent/caregivers, referred to the study by the professional staff, were selected because of their experience in caring for an adolescent taking HIV cART, without regard to adherence problems. The in-depth interviews for both the professionals and the caregivers were conducted in a private space located at a hospital in Lima, followed a semi-structured interview guide, were audio-recorded and lasted approximately 60 minutes.

The semi-structured interview guide content was determined by anecdotal information from previous interactions with professionals and caregivers of adolescents taking HIV cART as well as by relevant literature. Illustrative questions related to cART adherence that were asked of the professionals and caregivers are presented in Table 1.

**Table 1:** Illustrative cART adherence questions for in-depth interviews with professionals and parents/caregivers.

Audience	Question
**Professionals**	How would you describe your experience working with adolescents with HIV? What are some of the health problems or opportunistic infections with which adolescents most often present? How often do adverse reactions to ARV occur? Do some of the adolescents with HIV you know have problems with adherence?What types of problems and why? When are the adolescents hospitalized? Have you experienced a time when two or more adolescents with HIV help each other?
**Parents/Caregivers**	Has your adolescent learned to care for him/herself by, for example, taking his/her pills on time and eating well? What difficulties have you perceived that your son/daughter/charge has in managing his/her illness? Do you think that your son/daughter/charge knows to ask for help when s/he needs it? Would s/he be able to manage his/her own treatment? How do you help him/her take his/her ARV?

### Data analysis

Audio recordings from both the adolescent psychosocial support groups and the professional and parent/caregiver interviews were transcribed verbatim and loaded into the qualitative analysis software Dedoose [21]. Two researchers (MW and JG) first read the transcripts in their entirety to familiarize themselves with the data and to construct preliminary codebooks. Separate codebooks were created for the adolescent psychosocial groups and the professional and parent/caregiver datasets given the population and data collection differences. Next a systematic, comparative and descriptive content analysis was performed by re-reading each transcript and applying codes to thematically similar passages of texts. Though some codes were determined a priori, others were created de novo as new themes emerged. The text was then aggregated by each code and extracted into matrices to allow for the identification of recurring issues and differences in the narratives. Throughout the analysis, MW and JG met to discuss and resolve coding conflicts.

The analysis and conceptualization of the data were guided by social ecological systems theory to aid in understanding the reciprocal interplay between adolescents and their environment [22]. Once the data were extracted into matrices and consolidated into overarching themes andthose specifically related to cART adherence were mapped onto a SEM to better illustrate the findings.

### Compliance with ethical standards

Study materials were reviewed and approved by research ethics committees at the National Institute of Child Health (Instituto Nacional de Salud del Niño, INSN), Lima, Peru and Harvard Medical School, Boston, USA. For the psychosocial groups, we obtained written informed assent from adolescent participants and written informed consent from a parent or biological family member; in the case a caregiver was not a biological family member, we followed guidelines provided by the INSN ethics committee to obtain written informed consent from a legal guardian. For the in-depth interviews, written informed consent was obtained by the hospital professionals and parent/caregivers. Adolescent participants received a snack during the psychosocial groups and transportation stipends, and the professional staff and parent/caregiver participants were compensated for their time.

## Results

Table 2 summarizes the characteristics of the adolescents that participated in the psychosocial support groups. In all, 18 adolescents participated, nine in the 13–15 year-old group and nine in the 16–17 year-old group. All but two adolescents were infected with HIV perinatally: one by blood transfusion at seven months old and another whose infection route is unknown. Nearly all adolescents (15/18) were currently living with at least one family member, though half had a lifetime history of residence in a group home for children with HIV. Death of one or more of the adolescents’ parents was also common: 14/18 lost at least one parent to HIV/AIDS.

**Table 2:** Characteristics of adolescents that participated in the psychosocial support groups (N = 18).

Characteristic	n	13–15 year-olds(n)	16–17 year-olds(n)
**Sex**			
Female	10	6	4
Male	8	3	5
**HIV infection route**			
Perinatal	16	7	9
Blood transfusion	1	1	0
Unknown	1	1	0
**Currently living with/in**			
At least one biological parent	8	6	2
A non-parent family member	7	2	5
Adoptive parents	1	0	1
A non-family member	1	1	0
Group housing	1	0	1
**Lifetime history of residence in group home for children with HIV/AIDS**	9	2	7
**Death of at least one parent from HIV/AIDS**	14	5	9

Additionally, we conducted 15, individual, in-depth interviews, comprised of the five hospital professionals and the ten parents/caregivers. In all, the results are based on data extracted from the 24 group session transcripts (12 sessions for the 13–15 year-old group and 12 sessions for the 15–17 year-old group) and 15 individual interview transcripts.

**Fig 1:**
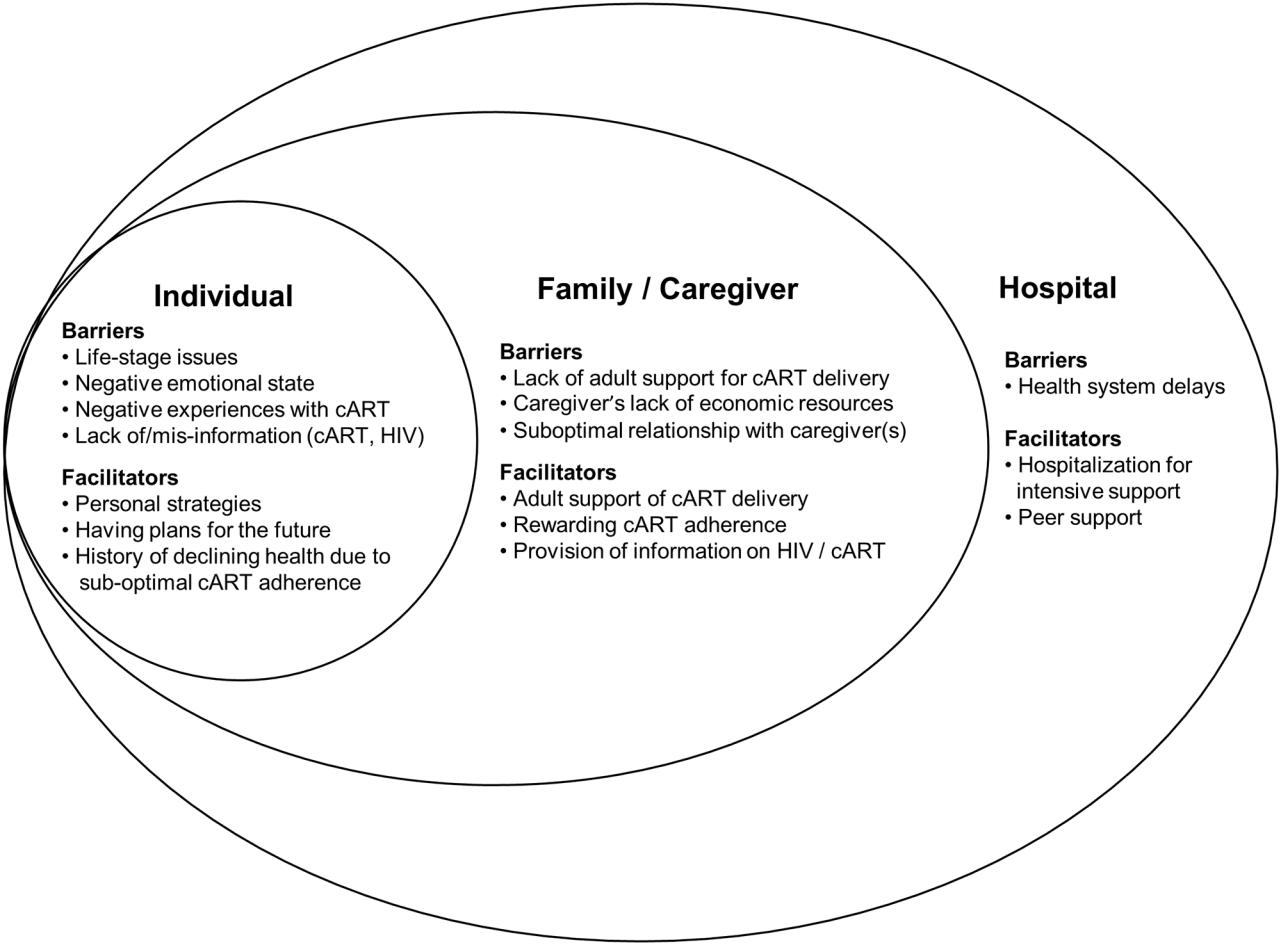
Social ecological model of barriers to and facilitators of antiretroviral cART adherence among Peruvian adolescents.

Barriers and facilitators to cART adherence were conceptualized following a SEM with individual (adolescent), family/caregiver and hospital levels (Fig 1). For each level, themes for barriers and facilitators are supported by illustrative quotes from the groups and interviews (see Table 3 for additional, supporting quotes and S1 Data for the unabridged qualitative data analysis). Participant names and other identifying information have been changed.

**Table 3:** Supplemental quotes for barriers and facilitators to cART adherence among Peruvian adolescents living with HIV at the individual, family/caregiver and hospital levels.

Level	Type	Theme	Quote
**Individual**	**Barrier**	Life stage issues	It’s a stage of changes: the child stops being a child and starts to be an adult, adolescent, and they feel tired of taking [their medication]. They fall in love, they get into other things and they forget to take their medicines. And it’s worse if there is no one on top of them like their fathers, grandparents or aunts/uncles who can remind them. They get argumentative: “I don’t want this anymore, I’m tired.”*- Administrative staff person*
		Negative emotional state	I want to keep my mother company and she is [in heaven].*- Luz*, *15*
		Negative experiences with cART	I felt that when I took a [cART] pill…Efavirnez…when I took it at night it made me feel…it didn’t make me laugh but it made me…gave me a buzz.*- Jorge*, *16*
		Lack of information/misinformation about HIV and/or cART	My questions would be about how to take the medicines at the exact hour, what to eat, tips, guiding me.*- Blanca*, *17*
	**Facilitator**	Personal Strategies	He has a pillbox where all his pills are sorted by week, so when it’s empty then every week he refills it and the pills are taken at 7:30 in the morning, 7:30 at night and 9 at night. That is the usual schedule.*- Caregiver*
		Having plans for the future	The first thing I want to do is soldier through this. So that’s two years and then I can have a new life. I want to get through this stage and then study mining engineering.*- Pablo*, *17*
		History of declining health due to sub-optimal cART adherence	I asked the doctor why they were changing the medicines, and I was told that they were being changed because I had stopped taking [my medication]. I had taken them for a week and then stopped for a month and that’s why my illness worsened, and they gave me stronger drugs to get better.- *Lourdes*, *15*
**Family/Caregiver**	**Barrier**	Lack of adult support	The parents say, “No, he’s already a grownup, I don’t have time” and they send them alone [to the hospital]. They say that they should get used to it, that they should get on with their things, and sometimes we must step in since the [public insurance program] won’t attend to them; they have to come with an adult.*- Administrative staff person*
		Caregiver’s lack of economic resources	If they could help me with the transportation cost; sometimes I don’t have [the bus fare]. For example [my child’s] dentist appointment is due and I don’t have the bus fare. And if I don’t work that day, who will pay for it? I don’t have anyone who can help. I don’t have other income.*- Mother of adolescent*
		Suboptimal relationship with caregiver(s)	Because he never understands me. He says that I am worthless, that he regrets that I was born. So, for that reason…that is when we began to argue.*- Carmen*, *17*
	**Facilitator**	Adult support	Moreover, since my sister always reminded me when to take [the medications], she always made sure that I had taken them, she says, “Did you take them?” Then later, I was living with my cousin, and she also heard the same thing and now she is also asking me, “Did you take them?” Now I don’t forget.*- Juan*, *17*
		Rewarding cART adherence	And they ask me, “How did we do?” And I tell them that we all got an A+ because everyone was undetectable. So that motivates them. And I also reward them, “You all behaved well so today we have this” and I’d ask them, “What do you want?”*- Group home caregiver*
		Provision of information on HIV and/or cART	The doctor told him why he had to take [the medication], and he asked for how long he had to take the pills. [The doctor] told him that he had to take them until they found a cure, and when they find something to cure you then you can stop taking [the pills].*- Grandmother of adolescent*
**Hospital**	**Barrier**	Health system delays	[I have been waiting for the medical chart] since last month when I came for the consult and now I came here and it’s not here. I have gone to the medical chart archive twice and they have not delivered it. Ever since I came here they have not yet brought it. I come in early since I have things to do. I organize myself so that they can see me and there’s no medical chart. Who’s responsible? If they were to give me the opportunity I would bring it myself and I’d be able to take better care of [my adolescent]…but it is not permitted.*- Grandmother of adolescent*
	**Facilitator**	Hospitalization for intensive support	We had an adolescent who had problems with his uncle and was homeless, so they wanted to hospitalize him since he had no place to go. So, if they’re sick, don’t have a home, we look to shelter them.*- Nurse*
		Peer support	Sometimes when the kids have problems at home, another [kid] who is in a better mood, who is more secure—more emotionally secure—is supportive [of the one having problems].*- Nurse*

### Individual level barriers

#### Life stage issues

The first barrier relates to how adolescents confronted life with the added burden of living with HIV and cART adherence. The adolescents in this study had questions regarding their lives in the context of living with HIV, particularly regarding forming romantic relationships or friendships with peers not living with HIV:

*They enter the period of falling in love, for example, “My girlfriend will find out what I am taking. She’s going to ask me why I am taking so many pills.” They say they can’t lie forever. They can say they have stomach problems…but it is the fear of being caught, so they abandon [cART]*.

Psychologist

Even going out with friends presented challenges, with the need to hide antiretrovirals so that friends would not see them:

*…so, I hid my medicines to be able to go out, to go with Laura and with Sara, and she noticed that I hid my medicines just to be able to go out*.

Carmen, 17

Facilitator: You’re not taking [your medication] on time, why is that?

*Milagros: Because I’m going out with my friends*.

Milagros, 14

#### Negative emotional state

Resentment and anger directed towards a parent for the adolescent’s HIV infection was also common and complicated the parent’s ability to ensure their child’s medication adherence:

*And when he turned 11, 12, 13 years old, he began to get up into my face telling me, “This is your fault. You ruined me, you took my life away.” He said to me, “Why did you take me to that hospital?” And from that point on I’ve been struggling [to get him to take his cART]*.

HIV-positive mother of adolescent

Finally, there were more generalized expressions of anxiety, intolerance and “boredom” with the prospect of cART being a lifelong requirement, as these adolescents lamented:

*Carlos: It ruins life*.

*Gustavo: Yeah, for sure it ruins life…it’s such a bore. Every day I have to remember, and that’s exactly why I can’t even go outside without [my] pills*.

Carlos, 13; Gustavo, 15

When probed further as to what stopping medication would mean to these adolescents, there was an overarching sense of hopelessness:

Facilitator: But if you’re already tired of the pills, then it’s because you want to die, is that it?

Luz: I do, yes…

*Gustavo: It just gets tiring, same for me and the pills*.

*Luz: I’m going to throw myself off a cliff*.

Facilitator: Who wants to die?

*Luz: Me*.

*Gustavo: Most everyone*.

*Carlos: Me*.

Luz, 15; Gustavo, 15; Carlos, 13

#### Negative experiences with antiretrovirals

Adverse reactions to the cART drugs used included feeling “drugged” or drowsy (or conversely having insomnia or feeling “energized”), bad flavor and nausea, vomiting or a “burning stomach” experienced after ingesting the medication:

*[The cART pills] were making me nauseous and from then on, I stopped taking my medicine*.

Luz, 15

Some adolescents spoke of multiple, combined side effects:

*I take Zidovudine, Abacavir, and Efavirenz and sometimes when Efavirenz keeps me up it makes me feel drugged. It makes me drowsy and it makes me dizzy during that period and I don’t sleep, and the next day I am affected, as well*.

Juan, 17

In this exchange, using the abbreviated expressions “Lami” and “Zido” to refer to antiretrovirals Zidovudine and Lamivudine, adolescents compare their experiences with the medication flavors:

*Facilitator: Lami tastes good, Zido is the one that gives you heartburn*.

*Gonzalo: Acidic, acidic. No, bitter…bitter*.

Carmen: And Kaletra?

*Giovana: It’s minty. It smells good but they say that it tastes horrible*.

Facilitator: What about you, how do you take it? Capsules or syrup?

*Carmen: Capsules. I hated the syrup because it burned everything. It was like [drinking] bleach*.

Gonzalo, 17; Carmen, 17; Giovana, 17

#### Lack of information or misinformation about HIV and/or cART

Lack of or incorrect information regarding HIV treatment was also a factor related to cART treatment adherence, beginning with the term “adherence” itself:

Facilitator: You all know what it means to be adherent, right?

Juan: No, but I suppose it’s something good?

Giovana: Adherence? I think it is…no, I don’t know…does it come from “inheritance”?

Juan, 15; Giovana 17

Evidence of misunderstanding about cART treatment indications also contributed to potential adherence barriers, as in the following example, where missed ARV doses were combined into a single dose:

*Gustavo: So, I take [the pills] in the morning before going to school. I take 13 pills… no, 14*.

*Luz*: *He takes pills*. *What he doesn’t take in the morning he takes at night*. *That’s what I used to do*.

Gustavo, 15; Luz, 15

In this example, medication that was prescribed to be taken at a specific hour was skipped altogether if not taken exactly on time:

Facilitator: You take your medication at 8 on the dot, and if you miss it?

*Carlos: Then I don’t take it*.

Gustavo: It’s not even worth it if it’s too late since it’s not going to count, it’s not going to count, and you’d be taking them for nothing, right?

Carlos, 13; Gustavo, 15

Despite having experienced the consequences of suboptimal cART adherence such as an illness requiring hospitalization, medical staff noted that a common knowledge gap among adolescents was why cART needed to be taken when they felt fine and were in good health:

[They often don’t understand that treatment] will never end, right? I mean, this is a treatment, and they always ask, “For how long? If I am healthy, why do I have to keep taking medication?”

Physician

### Individual level facilitators

#### Personal strategies

Adolescents developed multiple strategies to enhance cART adherence. Juan, for example, stores his medications in a conspicuous location at home, as a visual aid to help him remember to take them:

*I put them in my dresser, since that’s where I have my bathroom towel, that way I see [the medication] and I now I don’t forget to take them*.

Juan, 17

Another strategy mentioned by adolescents was the experimentation of different food and drink consumed with antiretrovirals to mask the flavor of the medication and increase its tolerability. In this psychosocial support group exchange, participants share examples of things they tried:

*Janet: [I take the medications] with a cappuccino*.

*Carolina: With water, fruit juice or yogurt*.

*Lourdes: With fruit juice or with yogurt*.

*Carlos: With boiled water*.

*Janet: When there’s a fruit that can be blended, [like] goldenberry, passion fruit, or with strawberry juice, papaya juice, strawberry with milk, or with milk alone. Or if I am eating and at the same time I take my pill, then I don’t feel nauseous*.

Janet, 15; Carolina, 14; Lourdes, 15; Carlos, 13

#### Having plans for the future

The second main cART adherence facilitator was adolescents’ plans for the future, for example studying for a professional, technical or occupational career:

*I want to try to do face painting since my dad and everyone else makes cakes and piñatas and everything for birthdays and I want to learn. When I stop taking [cART] I get sick, and I want to study to be a stylist and cosmetologist*.

Janet, 15

#### History of declining health due to suboptimal cART adherence

The third adherence facilitator was motivated by the fear of experiencing the negative health consequences of non-adherence, which could lead not only to hospitalization but could impact the realization of future plans. Janet continues,

*If stop talking [the medication], I get sick. One time I stopped taking my pills for five days and I got a fever, I lost weight and was admitted [to the hospital]. When I was discharged, I took my pills and I gained weight*.

Janet, 15

### Family/Caregiver level barriers

#### Lack of adult support for cART adherence

This barrier was raised both by adolescents who stated that cART adherence would be higher with adult support and by hospital staff who felt that treatment adherence was the parent/caregivers’ responsibility. Sometimes this support was related to reminding the adolescent to take the medication:

*They usually say that they forgot [to take their medication], and this happens most often when there is no one who is giving him the medication, because [the caregiver] says, “They’re grown, they are already adolescents, they can take the medications themselves.” But that is a mistake*.

Physician

*It’s that at night my mother forgets to give me [the medication] and I lose track of time [and forget to take them]*.

Mariela, 14

At other times, the support was related to the provision of other aspects of daily necessities, without which cART adherence was compromised, for example, having food prepared:

Facilitator: What time do you get home?

*Giovana: At 12*.

Facilitator: Why?

*Giovana: Because I work. And, well, sometimes I get home and my aunt is not there and so what am I supposed to do? I just go to bed. My aunt also gets home and goes to bed*.

Facilitator: And if there were dinner there? Then you’d take the pill?

*Giovana: Yes. One week I was doing that, but that’s because there was food that week (laughs)*.

Giovana, 17

#### Caregiver’s lack of economic resources

The lack of economic resources as a barrier to cART adherence was a common observation. This was especially in the case of adolescents with single mothers for whom the necessity of working to feed the family could translate into less vigilance regarding their child’s medication adherence. This mother, also living with HIV, explained her experience with her daughter and the challenges faced:

*There are times when the doctors would make me hysterical since they didn’t understand that I have to work. [They’d say,] “If you don’t come for your own health, then come first for your daughter.” [And I would say,] “Sure, but then where am I going to get the money for transportation?” Because I didn’t have the money and I wanted them to listen to me, understand that. And now I cannot work since then I can’t take care of Leyla, and Leyla doesn’t understand. I tell her that I must work and if you don’t take the medication, you get sick and then I stop working*.

HIV-positive mother of adolescent

#### Suboptimal adolescent-caretaker relationships

The parent/caretaker relationship could also affect cART adherence, either in cases of absent or disinterested caregivers or “overbearing” caregivers. In the case of adolescents with no stable family member looking after them, other medical problems apart from HIV could present:

*Apart from the HIV diagnosis, he has arthritis, psoriasis, and dermatitis due to zinc deficiency. I mean, he has 50 thousand diagnoses and on top of that he has depression, something for which really needs help since he has no stable family member who comes to see him. Nearly no one comes to visit*.

Physician

On the other end of the spectrum, a suboptimal parent/caregiver relationship affected adherence when adolescents rejected their parents’ concerns about their health. This parent explained her desperation:

*I don’t know if there is a solution at this point, to know what to say to her and be able to relate normally and not continue to be preoccupied with the pressure of saying “Have you taken [your medication]?” Or to be worried about how my daughter feels when I yell at her. I have yelled at her a lot and she told me she even wanted to die*.

HIV-positive mother of adolescent

### Family/Caregiver level facilitators

#### Adult support

While lack of adult support was a barrier to cART adherence, the opposite was also true: a parent or caregiver who was supporting their adolescent could have a positive impact on cART adherence. The support could be direct reminders or pill preparation, or it could be more subtle monitoring, as in the example provided by this mother:

*I can see how many [pills] he’s taking and where he’s taking them but I don’t leave it there. I’m watching television but keeping an eye on him; I’m watching to see how many how many he takes and how many he swallows*.

Mother of adolescent

#### Rewarding cART adherence

One adherence strategy employed by family/caregivers was rewarding cART adherence with things that the adolescent wanted:

My siblings told me, “We’ll help you with what you need. We’ve already bought a table.” But they say, “If I find out that you have not taken the pill, I’ll take it away from you and not help you with anything.”

Lourdes, 15

#### Provision of information on HIV, cART

Another adherence facilitator by family/caregivers was having information about HIV/cART which, in addition to explaining that the pills must be taken regularly at specific times, included information on the potential consequences of non-adherence in terms of what could happen with the immune system:

*[She told me that] I must take [the pills] on time, and if I don’t, then my CD4 [level] won’t rise. I have to get up early and keep at it since if I stop taking them for a day then that would be the same as if you were to lose 10% of your CD4+. She told me something like that*.

Pablo, 16

### Hospital level barriers

#### Health system delays

Caregivers and adolescents alike noted how a routine HIV care visit to the hospital, at which both the minor child and caregiver must be present, could last an entire morning or even a day thereby causing missed work (and wages) and schooling. Administrative issues such as missing clinical charts or long lines, as well as delays in public insurance paperwork, could also delay cART initiation. This adolescent describes, for example, how a paperwork delay when moving from one district to another resulted in her missing five months of cART treatment:

*What happened is that my care referral expired, and everything got delayed during which I went without medicine. They were able to provide some [medication] here for a while but later they couldn’t. My mom was doing the paperwork, but it didn’t get approved. That was for five months*.

Carolina, 14

### Hospital level sacilitators

#### Hospitalization for intensive support

Medical staff reported hospitalizing adolescents when more intensive support was needed. For example, if medical staff find that treatment adherence is low or there is an increase in viral load, they may opt to hospitalize the adolescent to provide intensive directly observed cART until the viral load is again undetectable. For example:

*We tell them that we will teach them how to take the medication and that we will hospitalize you. Many don’t want that, but later they accept it, and most accept it because they know that they have not being doing it well. They improve. Even the high viral loads drop totally. That means that they were fooling around and not taking their medication*.

Physician

#### Peer support

Hospital staff also felt that adolescents with high adherence to HIV cART often served as role models for other adolescents, helping them strengthen their cART adherence by talking about their experiences. These interactions often happened in the hospital environment where the adolescents would see each other during follow up visits and hospitalizations:

*They see other kids that have the same diagnosis. They see how they get on. And [these kids with high cART adherence] also many times have helped these kids to improve their adherence*.

Nurse

In addition to the self-identification with others living with HIV, adolescents also gave specific examples of providing support and encouragement to their peers during periods of hospitalization, including the necessity to take their medication:

*I talked to him, I said to him, real nicely I said to him to keep going and never stop taking your medication. And little by little he was doing that, taking them little by little, since when I saw him he wasn’t taking his medication or eating, not even eating, nothing. And later after I spoke with him then he started eating little by little and then he started to take his medication*.

Pablo, 16

*I was in a room here when I saw his sores. I saw him, and I said to him to get well, take your pills*.

Jorge, 16

## Discussion

Guided by a SEM mapped at the individual, family/caregiver and hospital levels, we identified barriers and facilitators to cART adherence among Peruvian adolescents. While barriers to cART adherence were abundant and included life-stage and emotional issues, negative experiences with cART, family and caregiver issues (support, relationships and economic issues), and structural issues at the care delivery level, there were also several cART facilitators which highlight the resilience, creativity and hope of many adolescents. Importantly, hospital level barriers were related to health system delays rather than, for example, inexperienced or unqualified staff, drug shortages or stigma. While hospital-based barriers should be addressed, as structural barriers that likely stretch beyond these adolescents’ HIV care to include care delivery in general at publicly funded/delivered health care services in resource restricted settings, changes may be difficult to implement. Instead, we identified several directly modifiable barriers that, along with the facilitators, could serve as the basis for tailored adherence interventions targeting adolescents and their family/caregivers.

Adherence to cART was at the heart of and often in conflict with the adolescent becoming an independent adult responsible for one’s health and self-care. During adolescence, parent involvement is often rejected as young men and women seek to establish their self-identities separate from their parents and caregivers [23]. As adolescents seek independence, social acceptance is also key, with a strong desire to "fit in." For some HIV-positive adolescents in this study, pill taking was perceived to jeopardize confidentiality, and they felt a need to choose between missing a cART dose or carrying their pills with them and risking disclosing their diagnosis. Taking cART was a reminder that they were not like other adolescents and it precluded them from participating in typical activities like spending time with friends and spontaneous social outings.

Relationships with other family members, especially parents/caregivers are often tested during adolescence, yet this is precisely the stage when parent/caregiver support is recognized as vital to optimal cART adherence. Both parents/caregivers and adolescents in our study noted this, and other studies report similar findings. For example, a study on cART adherence successes among HIV infected children in rural Uganda noted the importance of a “caregiver’s sense of obligation and commitment” as the factor potentiating adherence [14]. Likewise, a study on cART adherence among a sample of US adolescents found that children of caregivers who took responsibility for medicine ingestion were more likely to maintain adherence, with the authors concluding that “caregiver variables were so prominently predictive of adherence over time that caregiver interventions may be the first line for the prevention or treatment of nonadherence” [19]. However, multiple barriers conspired to undermine both the adolescent-caregiver relationship and the ability of caregivers to optimally support their adolescent’s cART adherence, including a parent’s need to work, parental death or stressed adolescent-parent relationships stemming back to birth when the child’s infection occurred. While addressing these complex issues may require special interventions, failure to do so may undermine the successful transition of the adolescent to adulthood, when cART adherence support from family may be limited or nonexistent.

Not all barriers were of a personal or interpersonal nature. The specific cART regimen composition and characteristics (pill size, number, medication taste) coupled with the need to take these medications for life also contributed to non-adherence, corroborating recent findings from a 2013 study in São Paulo, Brazil of 262 adolescents taking cART and of whom 50% reported difficulties with pill/capsule size and bad-tasting liquid medications [24]. While it is true that older (but effective) regimens or drug presentations likely contribute to unnecessarily high pill burdens, both our study and others point to a larger issue that unfortunately may never be resolved and is not unique to Peru: an overall lack of pediatric cART formulations available globally and market disincentives for developing cART regimens that are more palatable, easier to swallow and less complex to take [25]. Other alternatives, such as longer lasting cART (allowing fewer doses), and transdermal patches could also potentially boost cART adherence [26]. Likewise, Short Cycle Therapy (SCT) with long-acting drugs, which allow for regular, planned medication breaks (e.g., five days on medication / two days off) hold promise for boosting cART adherence in adolescents. [27]. Especially for adolescents who wish to keep their cART use private, having cART-free weekends could be attractive. [28] SCT, however, is still under investigation and has not been evaluated in Peru. In the meantime, interventions to support cART adherence need to work with the current reality for most adolescents—several adult-sized pills that taste bad—and find strategies such as those mentioned in this study to reduce the unpleasantness.

We found that information on HIV and cART or a personal experience with cART non-adherence facilitated adherence, while misinformation or lack of information was a key barrier both at the individual and family/caregiver levels. The psychosocial groups revealed that adolescents had a common, shared and erroneous understanding of basic medication instructions such as handling late or missed doses. Knowledge gaps regarding cART can lead to suboptimal adherence, even in adult populations. But simple, low-cost interventions such as graphically illustrated patient information leaflets may be effective at improving cART knowledge and self-efficacy and have the added benefit of not requiring high literacy levels [29]. A similar approach could be adapted for adolescents to graphically illustrate what cART does, how to take the pills and other related information (e.g., handling missed doses, not sharing pills, etc.). Such information would ideally be presented and used with both adolescents and their caregivers.

Our findings support the development and implementation of contextualized, multilevel interventions for adolescent cART adherence in Peru. Since most of the barriers to cART adherence clustered at the individual and family/caregiver levels, interventions that emphasize these levels are warranted. A similar approach has been reported in South Africa in the “VUKA Family Program,” which piloted a psychosocial program targeting HIV positive pre-adolescents and their caregivers. In this small study of 65 children and their caregivers, improvement on multiple dimensions was noted, including HIV treatment knowledge, stigma, communication and medication adherence [30]. We also note the likelihood that facilitators listed under the hospital level may relate more to the interventions received during hospitalization (e.g., directly observed cART administration; peer support from other adolescents living with HIV; nutritional support) than the act of hospitalization itself which would not be an ideal first line intervention for cART adherence. Moreover, these hospital-based cART adherence interventions could be provided in community-based settings if the appropriate programs and resources existed to support them.

While our data corroborate many of the findings from similar studies in other countries, there are also important differences. For example, stigma is often found to be a barrier to cART adherence [11,31] whereas in the present study, real or perceived stigma was presented primarily as a barrier to disclosure of HIV status rather than to cART adherence. We also did not find that alcohol or drug use nor mental illness (e.g. clinical depression, behavioral disorders) were barriers to cART adherence, again in contrast to similar studies [32–34]. Instead, we found that alcohol and drug use (cocaine and marijuana were mentioned) was discussed both in the psychosocial support groups and the in-depth interviews, but was uniformly characterized as brief experimentation unrelated to cART adherence. For mental health, while we identified “negative emotional states” as having a deleterious impact on adolescent cART adherence, clinically diagnosed mental illnesses were not singled out by hospital staff as a specific adherence barrier.

### Limitations

There were limitations to our study. First, the adolescents participated in psychosocial groups where session content was directed by the participants themselves rather than by a formal interview guide with planned questions and probes. Although the group facilitator asked probing questions to explore the chosen theme, the approach prevented a standardized structure to ensure that a range of topics was comprehensively discussed. However, a benefit to this methodology was that discussion topics emerged naturally without imposing the assumptions of investigators, an aspect which may be especially useful in working with adolescents where independence and self-determination is highly valued.

Next, the adolescents were not randomly chosen but prospectively selected for group participation due to their cART adherence challenges and were all receiving care at the same hospital in Lima, Peru. These adolescents were likely more apt to describe cART barriers than facilitators, and the facilitators that were described may be different than those experienced by adolescents living with HIV who do not have cART adherence challenges. We believe our findings are likely generalizable to perinatally infected adolescents in urban Latin American settings. But because nearly all adolescents were perinatally infected, the generalizability of these findings to behaviorally infected adolescents is unclear. Studies generally report similar adherence barriers among perinatally and behaviorally infected youth, though real or perceived stigma may be different between these groups [35]. The development of truly comprehensive adolescent HIV cART adherence interventions will need to include all adolescents, regardless of HIV infection route. In Peru, as in much of Latin America, HIV is concentrated in key groups (i.e., men who have sex with men and transgender women) with the highest incidence rates observed in youth less than 25 years old [36], adding extra relevance to this aspect.

Finally, we chose to facilitate our analysis using a framework (i.e. SEM) in which the central unit of analysis is the individual in relation to other social structures. As such, the medication-specific barriers (e.g., pill count, taste, side effects) were conceptualized from the perspective of the adolescents’ behaviors towards the medicines and may underemphasize the need for more pediatric-friendly cART options.

## Conclusion

Interventions to support adolescent HIV cART adherence are urgently needed and should be tailored to both the life stage and setting. In Peru, we found that most cART adherence barriers and facilitators clustered at the individual and family/caregiver levels—entry points that hold promise for adherence interventions. No adolescent living with HIV should die from AIDS in an era of accessible cART.

## Supporting information

S1 Data Full unabridged qualitative data analysis. (XLSX)
